# Quantitative Indicators of Microstructure and Texture Heterogeneity in Polycrystalline System

**DOI:** 10.3390/ma17246057

**Published:** 2024-12-11

**Authors:** Jurij J. Sidor

**Affiliations:** Eötvös Lorand University (ELTE), Faculty of Informatics, Savaria Institute of Technology, Karolyi Gaspar tér 4, 9700 Szombathely, Hungary; js@inf.elte.hu

**Keywords:** heterogeneity, microstructure, texture

## Abstract

The microstructural features of polycrystals determine numerous properties, whereas the evolution of crystallographic texture is responsible for the anisotropy of particular properties. Therefore, it is of crucial importance to find proper quantitative indicators, which reflect the nature of microstructure and texture characteristics. This is partially performed by the assessment of the average grain size and texture intensity that provide basic information on the microstructural features evolved; however, often, the basic quantitative indicators are not capable of revealing the complete microstructural state especially when the system is highly heterogeneous. This contribution presents various methods to assess the degree of microstructural heterogeneity, while the crystallographic aspect of microstructure evolution is characterized by several indicators of texture heterogeneity. Numerous synthetic microstructures with normal, lognormal, and bimodal grain size distributions as well as their combinations are analyzed to evaluate the applicability of the methods presented in this study. The quantitative indicators described in the frame of this contribution are likewise tested on experimentally observed microstructures. It is shown that the derived coefficients of microstructure heterogeneity correlate well with the standard deviation in grain size distribution, Gini, and Hoover coefficients, while the quantitative measures of texture heterogeneity are capable of revealing hidden aspects of microstructure evolution.

## 1. Introduction

Thermomechanical processing (TMP) of metallic materials accounts for complex microstructural transformations. The as-cast dendritic or equiaxed polycrystalline aggregates are subjected to further processing such as extrusion, rolling, forging, or other forming technologies. Each technology involves one or more characteristic strain modes while the resulting microstructure is strongly dependent on the degree of deformation, process temperature, processing time, and chemical composition of the material [1].

The changes in the deformation mode or temperature during the manufacturing process can be simulated by well-established numerical models such as the finite element model; however, the prediction of the microstructure evolution is not straightforward, since the development of polycrystalline assembly is a complex process and, in many instances, the kinetics of grain evolution is not identical for all constituents of the polycrystalline aggregates. As a result, heterogeneous structures tend to evolve.

Given the complexity of microstructure evolution, defining proper quantitative indicators for microstructure characterization is vital, and, therefore, the aim of this contribution is to analyze the quantitative measures, allowing the assessment of polycrystalline aggregates in terms of the microstructure homogeneity and heterogeneity of crystallographic texture.

### 1.1. Basic Characteristics of Microstructural Diversity

The vast diversity of microstructures evolved in different materials is typically characterized by optical microscopy, scanning electron microscopy, or electron backscattering diffraction [1,2,3,4,5]. These techniques enable revealing various aspects of microstructure evolution. In most general cases, the microstructures can be subdivided into the following morphological groups: (i) equiaxed and (ii) elongated. The first group is characteristic of the as-cast or recrystallized materials, while the second group is typically observed after deformations such as rolling or extrusion. A three-dimensional (3D) grain morphology can be revealed by inspecting the two-dimensional sections (2D). In the case of equiaxed structures, the 2D morphology will be nearly identical for any arbitrary section, while the elongated structure along one particular direction (extrusion or rolling direction) might reveal a completely different grain shape in the other two directions. Typical examples are needle-type, columnar, or pancake structures. The 2D morphology in the transverse direction plane (plane perpendicular to TD) will be elongated while the RD (reference direction) plane inspection will show an equiaxed (needle) or slightly elongated structure.

**Figure 1:**
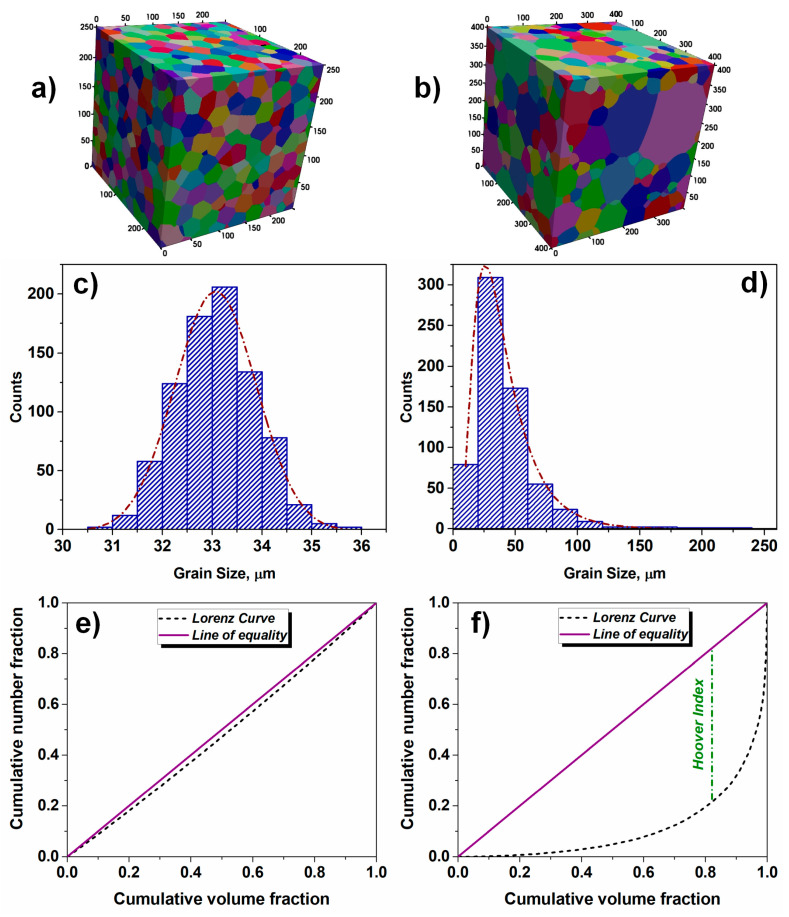
(**a**) Microstructure with homogeneous grain size distribution; (**b**) microstructure with significant grain size spread; (**c**) Gauss grain size distributions corresponding to Figure 1a; (**d**) lognormal grain size distribution corresponding to Figure 1b; (**e**) Lorenz curve computed for microstructure of Figure 1a; (**f**) Lorenz curve computed for microstructure of Figure 1b.

Apart from the morphological heterogeneity, the microstructure might be inhomogeneous in terms of grain size distribution (Figure 1). Even the most homogeneous polycrystalline aggregate reveals grain size diversity [6,7,8,9], which can be characterized by the Gauss probability function [10]:

(1) $$f\left(d\right)=A_{0}+Aexp(-\frac{{\left(d_{i}-d_{a}\right)}^{2}}{2{\left(std\right)}^{2}})$$

where *d_i_*—grain size, *A*_0_—offset, *d_a_*—average grain size, *std*—standard deviation, and *A*—amplitude that determines the height of the Gauss function.

The standard deviation is calculated as follows [10]:

(2) $$std=\sqrt{\frac{1}{N-1}\sum_{i}^{N}{\left(d_{i}-d_{a}\right)}^{2}}$$

where *N* is the total number of grains in the given representative volume element.

This type of distribution refers to the so-called normal distribution and is characterized by a symmetric bell-shaped scatter around the mean value. The Gauss-type function (see Figure 1a,c) perfectly describes microstructures with uniform grain sizes, where the values of d majorly cluster around the average value, while the number of counterparts away from the mean value tends to drop quite uniformly in both directions.

In many instances, equiaxed microstructures in single-phase alloys show lognormal size distribution [10]:

(3) $$f\left(d\right)=A_{0}+\frac{A}{(std)d_{i}\sqrt{2\pi }}exp(-\frac{{ln(d_{i}/d_{a})}^{2}}{2{\left(std\right)}^{2}})$$

The lognormal distribution resembles a distorted Gaussian curve, where the quantities rise from lower values towards a peak, and then gradually decline, creating a distinctive long tale (see Figure 1b,d). This type of distribution is characteristic of inhomogeneous grain assembly, where the length of the tail reflects the degree of heterogeneity. Both lognormal and Gauss distributions (Figure 1a–d) are quantified by the mean value and standard deviation; however, the comparison of these parameters, with the aim of revealing the degree of heterogeneity, is not straightforward since the two functions are different in nature. The comparison of cumulative probabilities of lognormal and other similar functions is summarized elsewhere [6]. It was concluded that the difficulty in choosing a proper distribution function is related to a high degree of experimental data scatter or poor statistics.

The next level of complexity, related to the characterization of microstructures, is given by the presence of bimodal grain size distribution [11]. This distribution can be deconvoluted into (i) two normal distributions with diverse mean grain sizes or (ii) lognormal and Gauss counterparts. In either case, it is not obvious how to assess the degree of heterogeneity, since the microstructure is inhomogeneous in nature but might consist of homogeneous counterparts (such as two Gauss distributions, shifted with respect to each other).

Determination of the representative grain size, which is characteristic of a given microstructure, is of key importance since this fundamental parameter is correlated to the mechanical and magnetic properties as well as the chemical behavior of materials [1]. The evaluation methods defined in National Standards [12,13] are more suitable for Gauss-type distributions, and the estimated value is close to the average grain size *d_a_*. For a given polycrystal, consisting of *N* grains, the average grain size *d_a_* can be defined as follows:

(4) $$d_{a}=\frac{1}{N}\sum_{i}^{N}d_{i}$$

Another approach enabling the estimation of grain size involves the analysis of the grain topology. Typically, the grain size is assessed by analyzing two-dimensional polished surfaces. The 2D grain features are transformed to three-dimensional grain attributes by employing stereological approaches [14,15,16,17,18,19,20,21]. Topological parameters offer insights into the division of grains to structural elements and their interconnections, regardless of the microstructural scale [14,15,16,17,18]. These quantities are dimensionless and do not depend on the scale of microstructures. Additionally, microstructural characteristics such as the surface area, volume fraction, and boundary or surface curvature can also be utilized to assess the grain size [19]. It should be underlined here that even the stereology of a simple cubic shape can be quite complex and depends on the planar section considered [20]. Given that grains are complexly shaped (polyhedral shapes in 3D), this further complicates the implementation of stereological approaches. This is particularly true when dealing with highly heterogeneous microstructures. The physically sound value of the representative grain size *d_r_* can be expressed in terms of volume fraction *w_i_* and grain size *d_i_* of each grain/crystal as follows:

(5) $$d_{r}=\sum_{i}^{N}w_{i}\;d_{i}$$

This expression is implemented in orientation image postprocessing software [22,23], used for microstructural visualization and analysis of texture datasets.

Another aspect of microstructure characterization is related to the assessment of microstructure heterogeneity. Numerous methods enable the description of the distribution of particles in materials. The so-called grid-based methodology is typically used for the estimation of the uniformity *U_f_* of particle distribution on the surface [24]. A similar approach can be employed for the assessment of microstructure homogeneity, by replacing particles with the grain boundary segments:

(6) $$U_{f}=\left(1-\frac{\sum_{i}^{N_{g}}\left|P_{i}-P_{a}\right|}{N_{g}P_{a}}\right)$$

*P_i_*—the number of grain boundary segments and *P_a_* is the average number of grain boundary segments in the grids, *N_g_*—grid number.

It should be noted here that grains in polycrystalline aggregates are of irregular shapes and the polished plane will always exhibit a certain size distribution even in the case of most homogeneous structures due to stereological features [21]. In other words, it is a challenging task to define a real grain size distribution from the two-dimensional observations, and, therefore, the assessment of grain boundary segments in a particular grid is somewhat arbitrary. In the case of a high degree of morphological orientation, the particular two-dimensional cross-sections might show significant similarity. In multi-phase grain assembly, both acicular and equiaxed grains will show distinct differences in various investigated cross-sections.

In image processing, the structural similarity index measure (SSIM) is employed for the quantification of diversity between an arbitrary and a reference image [25]. This quantitative measure can be used for evaluating local changes and/or similarities between the two investigated counterparts. However, this approach is inappropriate for revealing the quantitative differences between two polycrystalline systems inasmuch as the grain boundaries of two microstructures do not overlap (even in the case of most homogeneous microstructures, two neighboring volume elements will show different spatial distribution characters of grain boundaries). In economics and sociology, the equality of distribution of a given variable (for instance, income) is represented by the so-called Lorenz curve [26,27]. This curve is a graphical illustration of the cumulative percentages of the population over the cumulative shares of the variable and this dependence is used for revealing the inequality where the 45° line of perfect equality is correlated to the actual distribution. The degree of inequality is typically deduced from the shape of the Lorenz curve with respect to the line of perfect equality (see Figure 1). The greater discrepancy between the two curves accounts for the larger level of inequality. In this type of analysis, the quantitative measure of inequality is expressed in terms of the Hoover index (*HI*), which represents the greatest vertical difference between the Lorenz curve and the line of ideal equality. An alternative measure of inequality of wealth distribution among a group of people is expressed in terms of the Gini coefficient GI (or Gini index) [28]. The Gini index is computed as the ratio of the area between the Lorenz curve and the perfect equality line (45° line) to the area under the perfect equality line (see Figure 1e,f). Both *HI* and *GI* tend to range between 0 and 1, where the minimum resembles the ideally equal distribution, whereas the maximum represents an absolute inequality. The approach was already successfully used in the digital processing of signals [29,30,31]. The same measure of statistical dispersion can be derived for the description of microstructure homogeneity. By analogy with the methodology developed for signal processing [29,30,31], for the distribution of the volume fraction of grains *w_i_*, the mentioned quantities (*GI* and *HI*) can be computed as:

(7) $$GI=1-2\sum_{i=1}^{N}w_{i}\left(\frac{N-i+0.5}{N}\right)$$

(8) $$HI=0.5\sum_{i=1}^{N}\left|w_{i}-w_{a}\right|$$

where *w_a_* is the average volume fraction of grains (it should be noted here that *w_i_*-s must be sorted in ascending order).

By extracting the values of the *GI* and *HI* from 1 (modified Gini index GI* = 1 − *GI* and modified Hoover coefficient HI* = 1 − *HI*), one can estimate the degree of microstructure homogeneity, where 0 corresponds to zero homogeneity (heterogeneous polycrystalline aggregate) and the value of 1 accounts for an ideally homogeneous microstructure. In the present study, modified GI* and HI* will be used for the assessment of microstructures.

### 1.2. Crystallographic Diversities in Polycrystalline Aggregates

**Figure 2:**
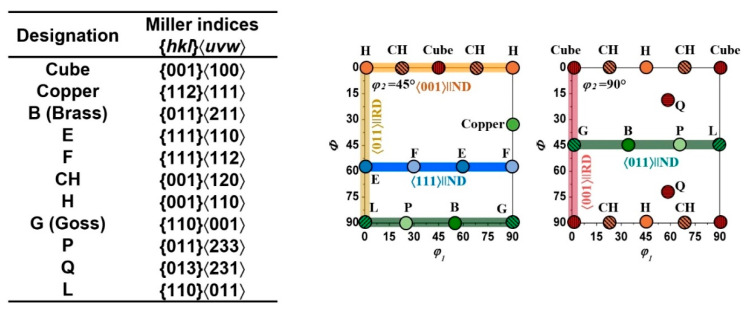
Main orientations and fibers in φ_2_ = 45° and φ_2_ = 90° ODF sections characterizing deformation and recrystallization textures in metals with FCC and BCC crystal structures.

In addition to the topological diversity, the polycrystalline systems are heterogeneous in terms of the evolution of crystallographic texture. The type of crystal structure, degree of deformation, stain mode, process temperature, and other thermomechanical processing parameters affect the development of texture in metals [1,32,33,34]. Typically, textures are represented in the form of orientation distribution functions (ODFs) in Euler space [1,35,36]. Random distribution of orientations in this space is quite rare and, in many instances, orientations are aligned along the so-called texture fibers, characterized by a common axis of orientations. The most common texture fibers, observed in metals with cubic crystal structures, are shown in Figure 2 [1,35,36]. In some instances, fibers have identical names but different common crystallographic axes of orientations. For example, the so-called α-fibers for FCC and BCC materials are 〈110〉//ND and 〈110〉//RD, respectively (ND and RD refer to normal and rolling directions) [1,34].

The degree of texture severity in materials, expressed in terms of texture index *TI*, is computed by integrating the squared values of orientation distribution function *f*(*g*) in Euler space [36]:

*TI* = ∫[*f*(*g*)]^2^*dg* (9)

Comparing particular ODF sections enables analyzing the evolution of textures, while revealing the degree of texture diversity is not straightforward and might be complicated due to a number of reasons. A qualitative comparison provides some insights about the distinguished features of textures evolved; however, choosing a proper set of quantitative indicators is of great importance. The difference between a reference ODF *f_ref_*(*g*) and any arbitrary texture *f_a_*(*g*) is typically accessed by the normalized texture index *TID_n_* [32,37,38]:

(10) $${TID}_{n}=\frac{\int {\left(f_{ref}\left(g\right)-f_{a}\left(g\right)\right)}^{2}dg}{\int {\left(f_{ref}\left(g\right)\right)}^{2}dg}$$

The normalized difference squared value between the two ODFs ensures a good idea about the quantitative measure of the texture diversity; nevertheless, this single value is not capable of showing all hidden aspects of texture varieties.

A scalar measure of texture heterogeneity *H_T_* [39] can reveal the degree of crystallographic diversity in a particular direction. This analysis assumes that the overall texture is composed of elements (columns or rows) characterized by different texture indexes. In this case, the degree of heterogeneity can be accessed by relating the average texture index of all elements $\bar{{TI}_{e}}$ to the overall *TI* [39]:

(11) $$H_{T}=\frac{\bar{{TI}_{e}}}{TI}-1$$

Equation (11) is suited for the characterization of planar texture varieties caused, for instance, by the so-called roping phenomena in metals [40,41].

The texture gradient severity *Θ* (degrees per unit of length) can be computed by the pole deviation function ω (average angular deviation of a considered pole for a particular reference direction) [42]:

(12) $$\Theta =\frac{1}{t}\int_{0}^{t}\frac{d\omega }{d\delta }d\delta$$

Here, *δ* represents a particular column in the scanned dataset corresponding to a specific depth along the cross-section [42].

## 2. Materials, Experimental, and Numerical Procedures

Numerous literature sources are dealing with the generation of 3D microstructures by analyzing 2D statistical distributions of polycrystalline aggregates [43,44,45,46,47]. The effort to create a comprehensive microstructural simulator from the experimental dataset has led to the development of an open-source software package (Dream3D (Version 6.5.160) [43,44]), designed for the generation and analysis of 3D microstructures. Dream3D program [43,44] provides a framework for generating representative volume elements of microstructures with predefined grain size distributions and specific grain morphological features as well as individual crystallographic textures. In this computational algorithm, the grain is approximated by an ellipsoid of corresponding volume *V_g_*. The equivalent grain diameter *d*_g_ is computed from the grain volume as follows [43,44]:

(13) dg=23Vg4π13

A spectrum of synthetic microstructures, analyzed in the present study, was generated by means of Dream3D software [43,44]. Microstructures of various average grain sizes, ranging between 12.2 and 163.9 μm, and diverse size distributions were produced and examined in terms of microstructure homogeneity. Normal, lognormal, and bimodal distributions cover the majority of metallic microstructures that tend to evolve in industrial practice, and, therefore, these grain assemblies were analyzed in detail. Both the size of the synthetic volume and the number of grains in the given box determine the period of computation. In order to decrease the computation time, the generated microstructures contain a reduced number of grains. Despite having quite a limited number of coarse grains in the corresponding bins of both lognormal and bimodal distributions, the generated grain assemblies were still used to make numerous sound conclusions about various distribution types. The size distribution parameters in the software are controlled via dispersion parameters. The experimentally observed grain aspect ratios (AR) and mean grain size as well as the distribution type are represented by the cumulative probability distribution [44]. The detailed mathematical descriptions of the microstructure simulation with predefined parameters are discussed in great detail elsewhere [43,44]. It should be underlined here that both normal and lognormal size distribution can be easily generated as single-phase polycrystalline systems, while the bimodal distributions can be reproduced by considering a polycrystalline aggregate as an assembly of two different microstructures with diverse mean sizes and different size distribution features.

In order to analyze Al-Mg-Si and Al-Mg alloys, the samples were subjected to electron backscattering diffraction (EBSD) scans across the thickness of the transverse direction plane. Prior to scanning, the mechanically polished samples were electropolished by using pre-cooled to 0–5 °C Struers A2^®^ electrolyte (Copenhagen, Denmark). The electrolytic polishing process lasted ~45 s, whereas the operating voltage was set to 30 V. A Hikari-type^®^ EBSD camera was attached to a high-resolution scanning electron microscope (SEM) FEI-Teneo (Thermo Fisher Scientific, Brno, Czech Republic), equipped with an FEG filament. During orientation imaging microscopy (OIM), the accelerating voltage of the SEM was set to 19 kV for optimal pattern capture. The sample was 70° tilted with respect to the EBSD detector, while the scans were performed on hexagonal grids.

For the post-processing of OIM data, the commercial OIM-TSL-8^®^ software (EDAX Inc., Mahwah, NJ, USA) was used. The calculated orientation distribution functions (ODFs) are displayed for the φ_2_ = 45°, φ_2_ = 65°, and φ_2_ = 90° sections. The ODFs were calculated with the MTMT software package [48].

In order to simulate the plastic strain ratio in the investigated 6xxx Al alloy (Al-Mg-Si), the advanced Lamel (ALAMEL) model was employed [37,38]. The {111} <110> family of slip systems was taken into account during the calculations of the Lankford value since they operate in materials with an FCC crystal structure during cold deformation [1,35].

## 3. Microstructure Heterogeneity: Theoretical Aspects of Quantitative Indicators

### 3.1. Description of Grain Groups

Both metallic and ceramic materials are polycrystalline aggregates (except for glassy materials); however, in many instances, the microstructure is heterogeneous in terms of the grain size. To better understand the nature of heterogeneity, the microstructure can be subdivided into a number of homogeneous groups (each group contains grains with nearly identical or similar sizes). Suppose we have a grain assembly of a volume *v*, which is composed of three groups as follows: (i) large *L* with the volume *v_L_*, (ii) middle *M* with the volume *v_M_*, and (iii) small *S* with the volume *v*_S_. The reciprocal value of the volume fraction of large grains (*Ω_L_*) is as follows:

(14) $$\Omega_{L}=\frac{v}{v_{L}}=\frac{\sum_{L}^{N_{L}}V_{L}+\sum_{M}^{N_{M}}V_{M}+\sum_{S}^{N_{S}}V_{S}}{\sum_{L}^{N_{L}}V_{L}}=1+\frac{{\bar{V}}_{M}N_{M}}{{\bar{V}}_{L}N_{L}}+\frac{{\bar{V}}_{S}N_{S}}{{\bar{V}}_{L}N_{L}}$$

where *N_X_* is the number of grains in an arbitrary group *X* and *V*_x_ is the volume of grain in group *X*.

By introducing the volume fraction *w* and number fraction *n* between grain groups as follows: $w_{IJ}=\frac{{\bar{V}}_{I}}{{\bar{V}}_{J}}$ and $n_{IJ}=\frac{N_{I}}{N_{J}}$, Equation (14) gains the following form:

(15) $$\Omega_{L}=1+w_{ML}n_{ML}+w_{SL}n_{SL}$$

For the middle and small grain groups, the corresponding *Ω_M_* and *Ω_S_* are the following:

(16) $$\Omega_{M}={\left(w_{ML}n_{ML}\right)}^{-1}+1+w_{SM}n_{SM}$$

(17) $$\Omega_{S}={\left(w_{SL}n_{SL}\right)}^{-1}+{\left(w_{SM}n_{SM}\right)}^{-1}+1$$

Equations (15)–(17) can be rewritten in the matrix form, where ***Ω_L_*** = ***Ω*****_11_** *= **Ω*****_1_, *Ω_M_*** = ***Ω*****_22_** = ***Ω*****_2_**, and ***Ω_S_*** = ***Ω*****_33_** = ***Ω*****_3_** are diagonal elements of the ***Ω*** matrix:

(18) $$\Omega =\left(\begin{array}{ccc} 1 & w_{ML} & w_{SL}\\ w_{LM} & 1 & w_{SM}\\ w_{LS} & w_{MS} & 1 \end{array}\right)\left(\begin{array}{ccc} 1 & n_{LM} & n_{LS}\\ n_{ML} & 1 & n_{MS}\\ n_{SL} & n_{SM} & 1 \end{array}\right)$$

In the most general case, when the system consists of *U* grain groups and the groups are sorted in the descending sequence (index 1 refers to the largest grains in terms of grain size, while index *U* corresponds to the finest g):

(19) $$\Omega =\left(\begin{array}{ccc} 1 & w_{21} & \begin{array}{ccc} w_{31} & \;\ldots & w_{U1} \end{array}\\ w_{12} & 1 & \begin{array}{ccc} w_{32} & \;\ldots & w_{U2} \end{array}\\ \begin{array}{c} w_{13}\\ .\\ w_{1U} \end{array} & \begin{array}{c} w_{23}\\ .\\ w_{2U} \end{array} & \begin{array}{c} \begin{array}{ccc} 1 & \;\;\;\;\;\;\ldots & w_{U3} \end{array}\\ \begin{array}{ccc} . & \;\;\;\;\;\;\;\ldots & \;\;\;. \end{array}\\ \begin{array}{ccc} w_{3U} & \ldots & 1 \end{array} \end{array} \end{array}\right)\left(\begin{array}{ccc} 1 & n_{12} & \begin{array}{ccc} n_{13} & \ldots & n_{1U} \end{array}\\ n_{21} & 1 & \begin{array}{ccc} n_{23} & \ldots & n_{2U} \end{array}\\ \begin{array}{c} n_{31}\\ .\\ n_{U1} \end{array} & \begin{array}{c} n_{32}\\ .\\ n_{U2} \end{array} & \begin{array}{ccc} 1 & \ldots & n_{3U}\\ . & \ldots & .\\ n_{U3} & \ldots & 1 \end{array} \end{array}\right)$$

The diagonal elements of the ***Ω*** matrix (***Ω*_11_** = ***Ω*_1_**;***Ω*****_22_** = ***Ω*_2_** …***Ω_UU_*** = ***Ω_U_***) are of particular importance since they represent the inverse of the volume fraction of a particular grain group *Λ* (1 ≤ *Λ* ≤ *U*). The following expression is valid for the diagonal elements ***Ω_Λ_***:

***Ω_Λ_* = *Ω_ij_δ_ij_*** (20)

where ***δ_ij_***is a Kronecker delta function.

The ***Ω_Λ_*** elements represent arbitrary grain groups and can be directly employed for the calculation of GI* and HI* quantitative indicators:

(21) $${GI}^{*}=2\sum_{\Lambda =1}^{N}\frac{1}{\Omega_{\Lambda }}\left(\frac{N-\Lambda +0.5}{N}\right)$$

(22) $${HI}^{*}=1-0.5\sum_{\Lambda =1}^{N}\left|\frac{1}{\Omega_{\Lambda }}-{\;w}_{a}\right|$$

In the case of an idealized homogeneous microstructure, composed of identical grains, there will be one grain group with *Ω_1_* = 1. However, real microstructures always show grain size spread and in the case of Gaussian distribution (see Figure 1a), the grain assembly can be subdivided into two groups in the following way: (i) two groups of nearly identical volume fractions and (ii) two groups of identical grain numbers. In the first case, *w*_12_ ≈ *w*_21_ ≈ 1, *n*_12_ = 1.1, and *n*_21_ = 0.9, while in the second case, *n*_12_ = *n*_21_ = 1, *w*_12_ = 1.1, and *w*_21_ = 0.9. In both considered cases, the elements of the ***w*** and ***n*** matrixes are close to unity, and this might be considered as an indicator of homogeneity. However, when the microstructure is characterized by the lognormal distribution (see Figure 1b), the same subdivision will account for the significant deviation of *w_ij_* and *n_ij_* from 1. For the distribution of Figure 1b with nearly identical volume fractions of both grain groups, *w*_12_ ≈ *w*_21_ ≈ 1, *n*_12_ = 28.9, and *n*_21_ = 3.46 × 10^−2^, whereas if *n*_12_ = *n*_21_ = 1, *w*_12_ = 5.18 × 10^−2^ and *w*_21_ = 19.32. Analyzing these two cases, it becomes obvious that the volume fraction of grains and their number can be used as indicators of microstructure homogeneity.

### 3.2. Assessment of Microstructure Homogeneity

In view of the above analysis, it follows that the degree of homogeneity is affected by the volume fraction of considered grain groups and the number of grains in each group. Given this, it seems that the relation of average grain size *d_a_* to the representative counterpart *d_r_* (see Equations (4) and (5)) can be employed for the quantitative description of microstructure homogeneity *H_d_*:

(23) $$H_{d}=\frac{d_{a}}{d_{r}}$$

If the material with a total volume *V* consists of *N* identical grains (*d_i_* = const ⇒ *V_i_* = const), the *H_d_* will be equal to 1:

(24) $$H_{d}=\frac{\frac{1}{N}\sum_{i}^{N}d_{i}}{{\sum_{i}^{N}w_{i}d}_{i}}=\frac{d_{i}}{{d_{i}\sum_{i}^{N}w}_{i}}=1$$

**Figure 3:**
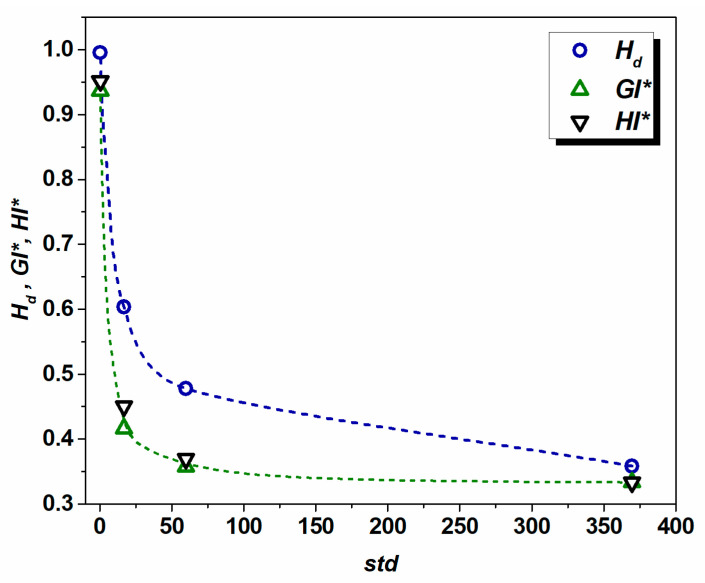
Dependence of *H_d_*, GI*, and HI* over standard deviation for grain assemblies with different degrees of heterogeneity (see text for details).

Let us consider four hypothetical cases when the representative volume element of a microstructure is subdivided into three homogeneous grain groups with the identical number of grains (*N*_1_ *= N*_2_ *= N*_3_ *=* 10 ⇒ *N* = 30): (1) *d*_1_ *=* 10 μm, *d*_2_ = 10.5 μm, *d*_3_ = 11 μm; (2) *d*_1_ *=* 10 μm, *d*_2_ = 25 μm, *d*_3_ = 50 μm; (3) *d*_1_ = 10 μm, *d*_2_ = 50 μm, *d*_3_ = 150 μm; and (4) *d*_1_ = 10 μm, d_2_ *=* 50 μm, *d*_3_ = 800 μm. The calculated degree of homogeneity/heterogeneity *H_d_* and standard deviation *std* for the above cases show meaningful qualitative correlation: *H_d_ (1)* = 0.996, *std (1)* = 0.42; *H_d_ (2)* = 0.603, *std (2)* = 16.78; *H_d_ (3)* = 0.478, *std (3)* = 59.88; and *H_d_ (4)* = 0.358, *std (4)* = 369.56. It is evident that in the present simplified scenarios, the value of *H_d_* tends to drop with the increase in heterogeneity (see Figure 3). The same evolutionary pattern is observed for the GI* and HI* quantitative measures of homogeneity. This suggests that the proposed method effectively reflects the level of heterogeneity. The higher value of *H_d_* corresponds to the higher degree of homogeneity and vice versa.

In view of the three-dimensional characteristics of grain assembly, the degree of homogeneity can be calculated by the following approach:

(25) $$H_{v}=\frac{\frac{1}{N}\sum_{i}^{N}V_{i}}{{\sum_{i}^{N}w_{i}V}_{i}}$$

If the equiaxed microstructure consists of identical grains (axes of the grain, approximated by ellipsoid are as follows: *a_i_ = b_i_ = c_i_* = *d* ⇒ *V_i_ = const*; *w_i_ = const*), the value of *H_v_* will be equal to 1:

(26) $$H_{v}=\frac{\frac{1}{N}\sum_{i}^{N}{{{\frac{4}{3}\pi a}_{i}b}_{i}c}_{i}}{\sum_{i}^{N}{{{{\frac{4}{3}\pi w}_{i}a}_{i}b}_{i}c}_{i}}=\frac{d^{3}}{d^{3}\sum_{i}^{N}w_{i}}=1$$

In the case of homogeneous microstructures, when all grains are identical and elongated in one particular direction (*a_i_ = Pb_i_ = Qc_i_* = *const*, while *P* and *Q* are constant values ⇒ *V_i_ = const*; *w_i_ = const*), the value of *H_v_* will likewise be equal to 1:

(27) $$H_{v}=\frac{\frac{1}{N}\sum_{i}^{N}{{{\frac{4}{3}\pi a}_{i}b}_{i}c}_{i}}{\sum_{i}^{N}{{{{\frac{4}{3}\pi w}_{i}a}_{i}b}_{i}c}_{i}}=\frac{\frac{1}{NPQ}\sum_{i}^{N}a_{i}^{3}}{\frac{1}{PQ}\sum_{i}^{N}w_{i}a_{i}^{3}}=\frac{{Na}_{i}^{3}}{Na_{i}^{3}\sum_{i}^{N}w_{i}}=1$$

**Figure 4:**
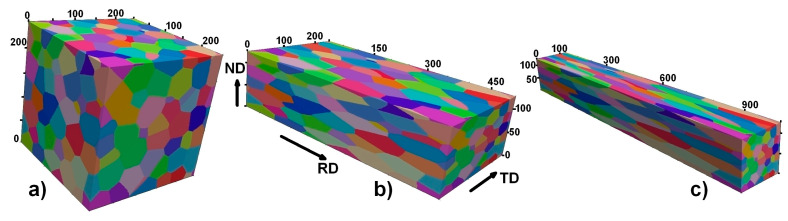
Homogeneous microstructures: (**a**) equiaxed (*H_d_* = 0.998, *H_v_* = 0.994, GI* = 0.958, HI* = 0.971, *std* = 1.36, *d_a_* = 54.5 μm; *d_r_* = 54.6 μm); (**b**) rolled with 50% thickness reduction (*H_d_* = 0.998, *H_v_* = 0.994, GI* = 0.957, HI* = 0.970, *std* = 1.37, *d_a_* = 54.5 μm; *d_r_* = 54.6 μm); (**c**) extruded with 50% thickness and width reduction (*H_d_* = 0.998, *H_v_* = 0.994, GI* = 0.958, HI* = 0.971, *std* = 1.34, *d_a_* = 54.5 μm; *d_r_* = 54.6 μm).

Depending on the thermomechanical processing, the microstructure might be composed of grains of various aspect ratios. Figure 4 shows the shape (morphological) transformation caused by two different deformation modes. Virtual plane strain compression (PSC or rolling) with 50% thickness reduction and virtual extrusion with identical reduction in both the width and thickness tend to produce diverse microstructures. In the case when no grain fragmentation is taken into account, the uniform scaling down of sizes along the normal direction can be performed according to the topological theory of grain deformation [49]. As Figure 4 reveals, the initially equiaxed (AR ≈ 1) homogeneous microstructure ensures a comparable degree of homogeneity in the deformed state (see Figure 4a–c). The representative grain aspect ratios observed in the planes perpendicular to the transverse and reference directions (direction of material flow) are ~4 and ~2, respectively, whereas for the extrusion, the ARs for the mentioned planes are ~8 and ~1. Although the resulting grain aggregates (Figure 4b,c) show diverse aspect ratios, the calculated values of *H_v_* and *H_d_* (*H_v_* ≈ *H_d_* ≈ 1) indicate that these quantitative indicators/measures are not sensitive to the morphological (grain shape) changes and capable of assessing the degree of heterogeneity in microstructures of diverse grain shapes. The high degree of homogeneity is additionally proven by the computed GI* and HI* indexes (see Figure 4 for details). It should be noted here that in severely deformed metals, the phenomenon of grain fragmentation will take place and the examination of the degree of heterogeneity might help in revealing the true nature of deformation.

**Figure 5:**
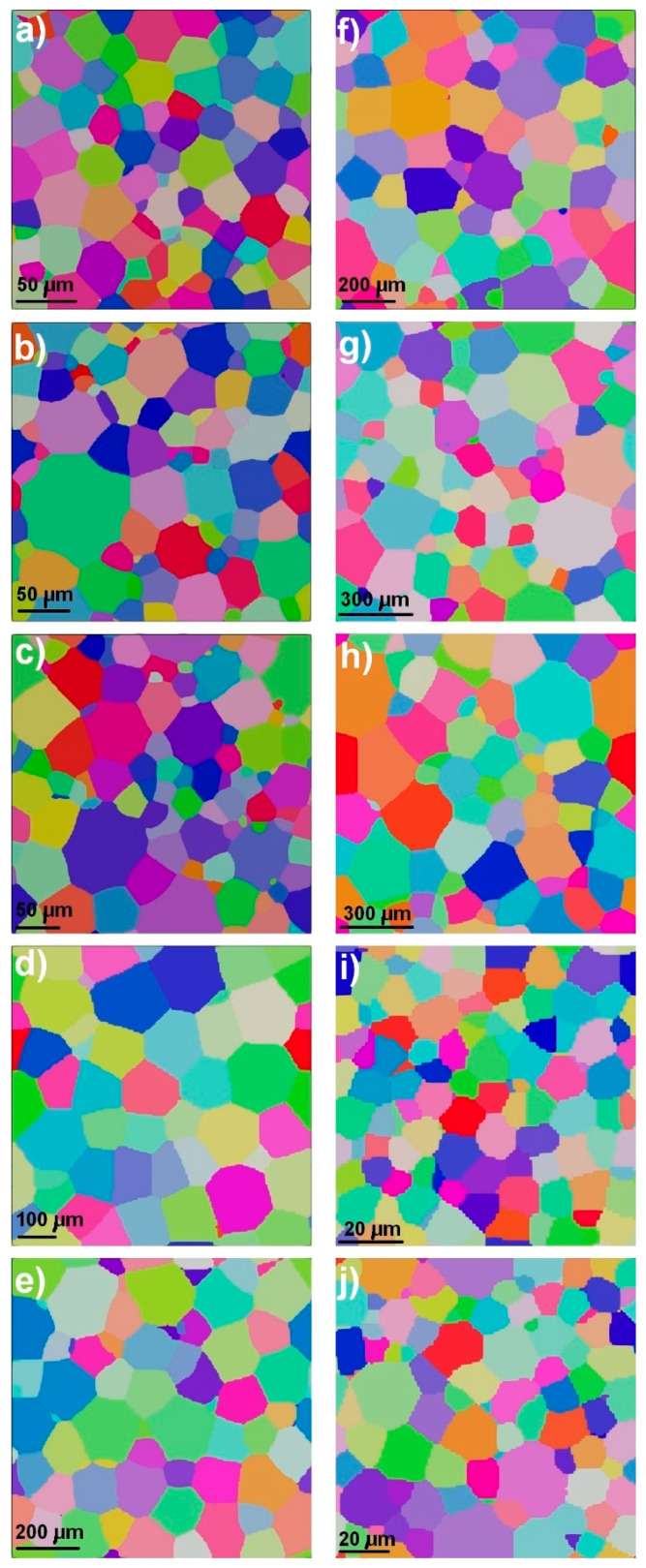
Synthetic microstructures of diverse grain size distribution: (**a**) d_a_ = 33.7 μm; (**b**) d_a_ = 36.4 μm; (**c**) d_a_ = 37.5 μm; (**d**) d_a_ = 149 μm; (**e**) d_a_ = 150.8 μm; (**f**) d_a_ = 154.5 μm; (**g**) d_a_ = 159.1 μm; (**h**) d_a_ = 163.9 μm; (**i**) d_a_ = 12.2 μm; (**j**) d_a_ = 13.6 μm.

**Figure 6:**
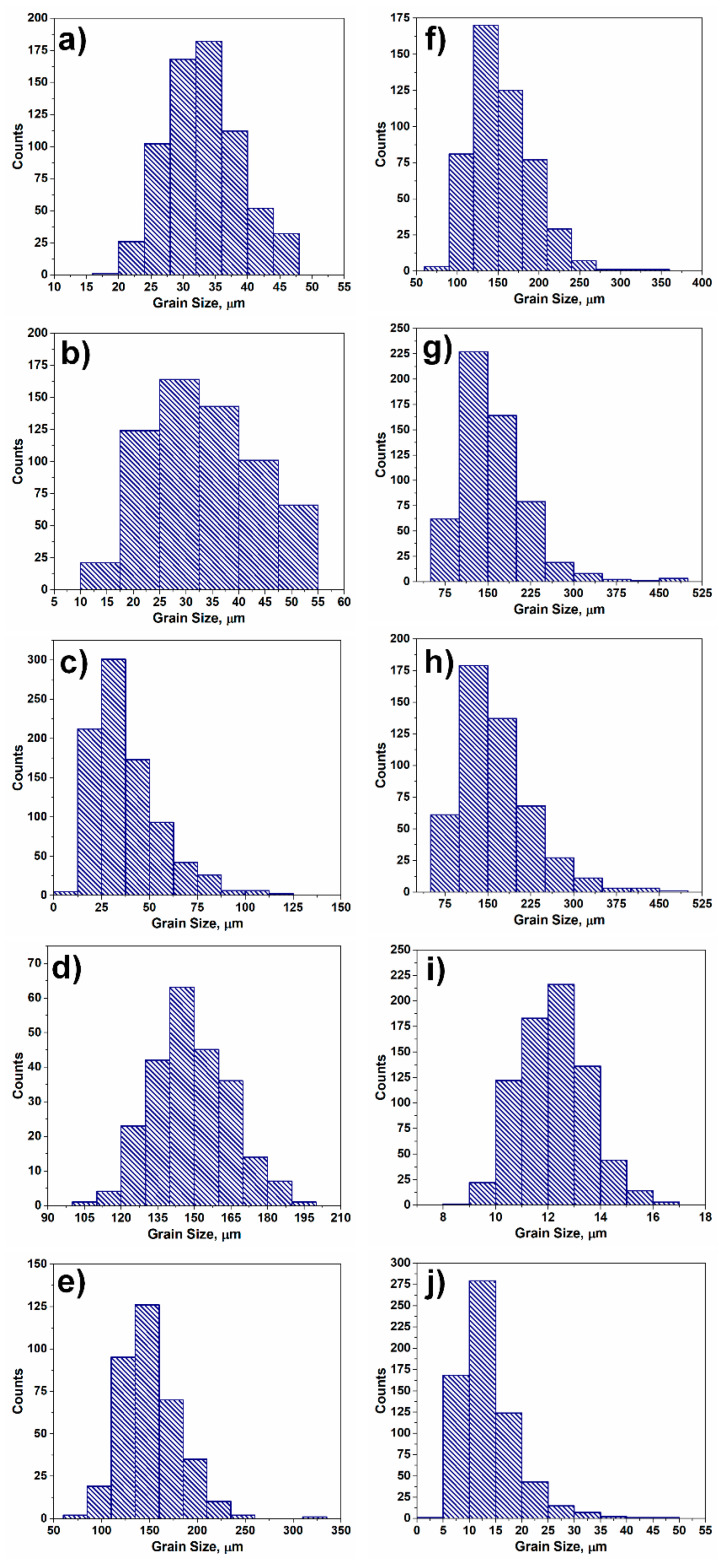
Grain size distributions of grain assemblies presented in Figure 5: (**a**) d_a_ = 33.7 μm; (**b**) d_a_ = 36.4 μm; (**c**) d_a_ = 37.5 μm; (**d**) d_a_ = 149 μm; (**e**) d_a_ = 150.8 μm; (**f**) d_a_ = 154.5 μm; (**g**) d_a_ = 159.1 μm; (**h**) d_a_ = 163.9 μm; (**i**) d_a_ = 12.2 μm; (**j**) d_a_ = 13.6 μm.

The microstructures shown in Figure 1a and Figure 4 are homogeneous in terms of the grain size; however, the polycrystalline aggregates can also reveal greater degrees of heterogeneity. Given this complexity, it is reasonable to analyze microstructures with different grain sizes and size distributions. Figure 5 shows twelve generated microstructures with distinct grain sizes and diverse size distributions (see Figure 6), while Table 1 shows the computed quantitative indicators for the generated polycrystalline systems. By analyzing the dependence of standard deviation on both *H_v_* and *H_d_* for the coarse, intermediate, and fine-grained materials, one can see that there is no clear correlation between the proposed quantitative measures of heterogeneity and the *std*. The major reason why it is difficult to attribute the standard deviation to the degree of homogeneity is that the value of *std* is grain size-dependent.

For the microstructure with the average grain size of 149 μm, the calculated *std* is 15.28, while for the counterpart with the *d_a_* of 12.2 μm, *std =* 1.29 (see Figure 5d,i). By comparing the calculated *std* values and analyzing the microstructures of Figure 5d,i, it becomes obvious that *std* solely does not provide complete information about microstructure homogeneity. The computed values of *H_d_* and *H_v_* in both cases are comparable (*H_d_* ≈ 0.97, *H_v_* ≈ 0.91), suggesting that the corresponding microstructures reveal a high degree of homogeneity. The high degree of homogeneity for these two microstructures is equally revealed by the calculated GI* and HI* coefficients (Table 1).

**Figure 7:**
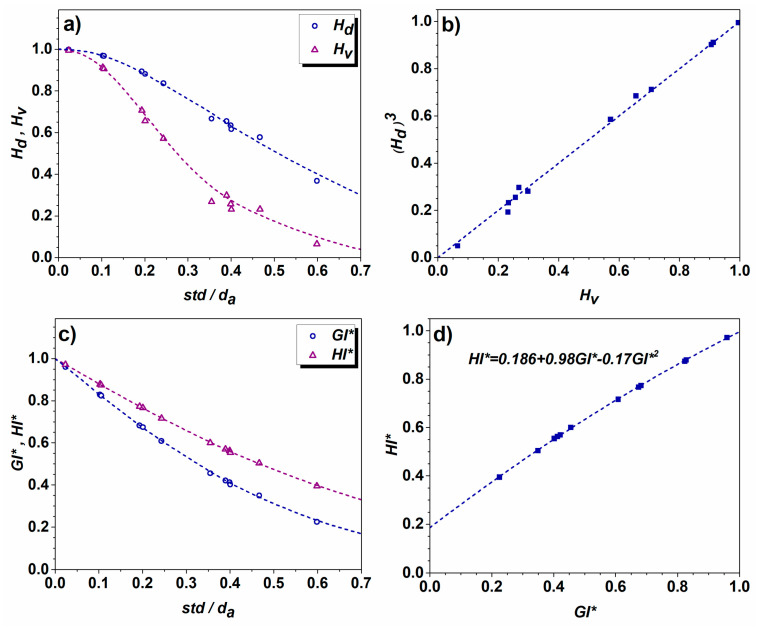
Correlation between the microstructure heterogeneity quantitative indicators for Gauss-type and lognormal distributions: (**a**) dependence of *H_d_* and *H_v_* on standard deviation normalized with the average grain size; (**b**) linear relationship between *H_d_*^3^ and *H_v_*; (**c**) dependence of GI*and HI* on standard deviation normalized with the average grain size; (**d**) polynomial relation between HI* and GI*.

By normalizing *std* with *d_a_*, a clear trend is observed among the quantitative indicators of homogeneity (Figure 7a). It seems that *H_v_* better reflects the nature of heterogeneity, since in the case of grain assembly with the largest grain size diversity (maximum to minimum grain size ratio d_max_/d_min_ = 23.3), the computed value of *H_v_* is 0.066, whereas *H_d_* is 0.368 (the microstructure is presented in Figure 1b). The ratio of d_max_/d_min_ cannot reflect the true nature of heterogeneity since in the case of relatively homogeneous microstructures, one grain of minimum size out of a million might lead to a wrong conclusion. Since the correlation between the volume and grain size is *V~d^3^*, a linear dependence between the *H_v_* and (*H_d_*)^3^ is observed (Figure 7b). The GI* and HI* indicators of homogeneity also tend to drop with an increase in normalized standard deviation (see Figure 7c), while the correlation between the two quantities can be described by polynomial relation (see Figure 7d). It can be concluded here that the newly proposed quantitative measures *H_v_* and *H_d_* are suitable for the assessment of microstructure homogeneity. In the case of highly heterogeneous polycrystalline assembly, the grain volume-based homogeneity indicator *H_v_* approaches zero faster, compared to *H_d_*, inasmuch as *V~d^3^*, *H_v_*~(*H_d_*)^3,^ and *H_d_* ≤ 1. The major advantage of employing *H_v_* and *H_d_* is that the data should be neither assigned to particular groups nor sorted in ascending order.

The degree of microstructure uniformity (homogeneity) can be performed by employing Equation (6). In the present study, the value of *U_f_* was calculated for two extreme cases: microstructures shown in Figure 1a,b (highly homogeneous and highly heterogeneous grain assemblies). The uniform grids of 25 × 25 μm and 40 × 40 μm were used for the analysis of two-dimensional (2D) slices, for the homogeneous and heterogeneous microstructures, respectively. In both cases, the cell sizes were smaller than the corresponding average grain size (see Table 1). In the case of the homogeneous microstructure of Figure 1a, the computed value of *U_f_* is 0.75, whereas *H_v_*, *H_d_*, GI*, and HI* are close to 1 (see Table 1). For the inhomogeneous microstructure of Figure 1b, the computed value of *U_f_* is 0.28, while all employed quantitative indicators of homogeneity show low values (see Table 1), suggesting that the grain size distribution reveals a wide spread. There is a good qualitative correlation between the estimated indicators of homogeneity; however, it is obvious that the proposed quantitative indicators *H_v_*, *H_d_*, GI*, and HI* better reflect the extent of microstructure homogeneity, compared to *U_f_*. The level of uniformity *U_f_* = 0.75 suggests that the microstructure of Figure 1a is relatively homogeneous with certain elements of heterogeneity; however, a more detailed analysis of quantitative indicators (see Table 1) provides evidence characteristic of an almost ideal homogeneous assembly. Therefore, the uniformity indicator is not capable of reflecting the complete nature of microstructure homogeneity.

**Figure 8:**
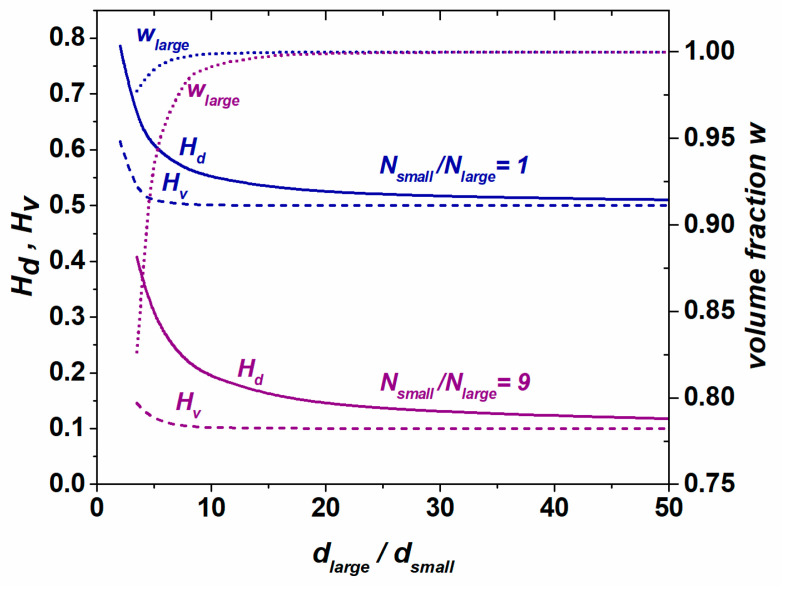
Correlation among the microstructure quantitative indicators, grain size ratio, grain number ratio, and volume fraction of large grains in microstructures, characterized by bimodal size distribution.

Figure 8 presents the dependencies of the microstructure quantitative indicators over the grain size ratio and volume fraction of large grains for the grain assemblies consisting of two ideal grain groups. In the studied hypothetical cases, each group was composed of identical grains (*H_d_*(large) = *H_v_*(large) = 1; and *H_d_*(small) = *H_v_*(small) = 1). Figure 8 reveals that *H_d_* and *H_v_* are dependent on the following parameters: (i) the grain size ratio of large to small grains *d_large_*/*d_small_*; (ii) the grain number in either group; and (iii) the volume fraction of grains in each group. It turns out that the grain number ratio has a decisive influence on the degree of microstructure heterogeneity: the large value of *N_small_/N_large_* ensures a greater degree of heterogeneity. It is important to note that even when the volume fraction of large grains tends to approach the value of 1, both quantitative indicators still show a high degree of heterogeneity, and this is rational since the microstructure is composed of differently sized grains.

**Figure 9:**
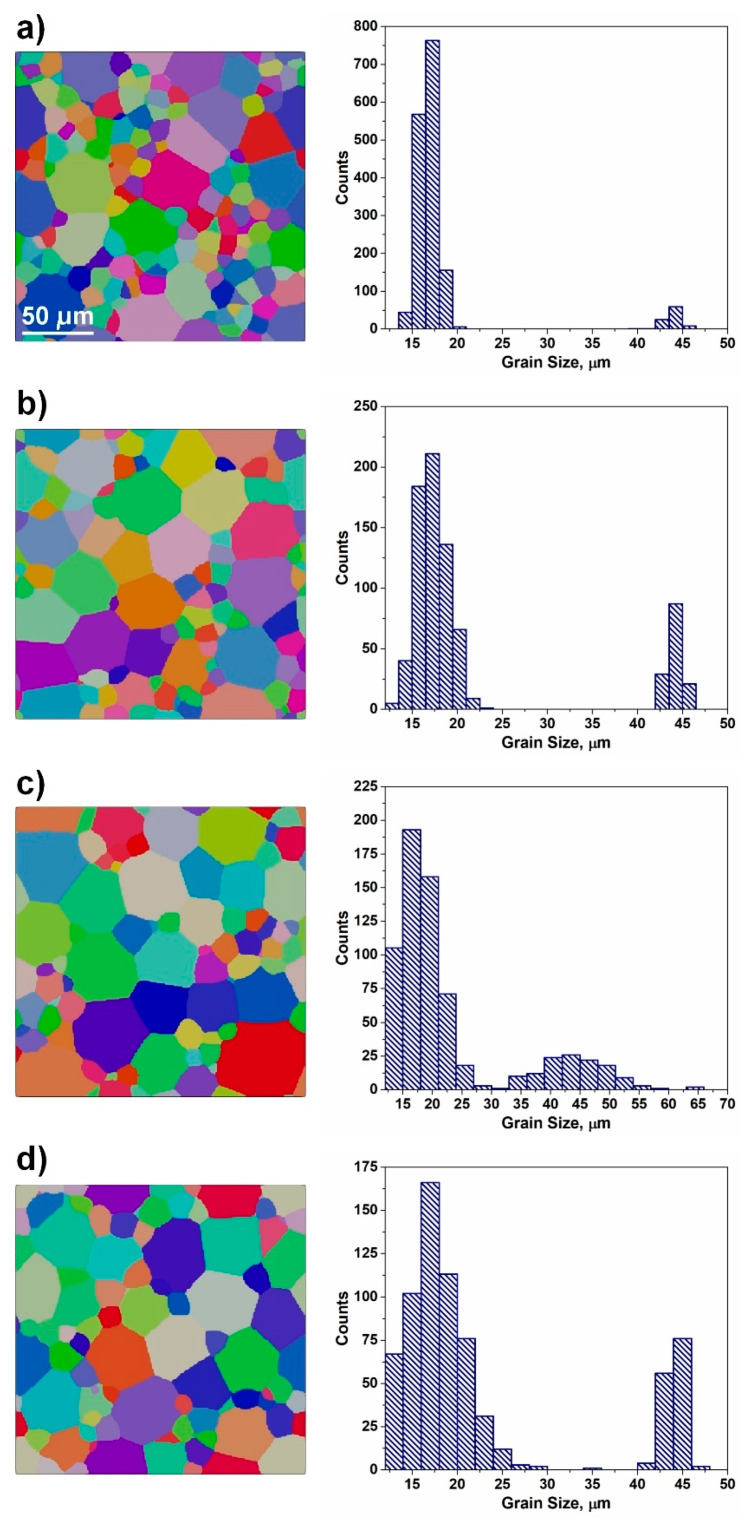
Various bimodal microstructures with corresponding grain size distributions: (**a**) **Bimodal1**: two Gauss-type distributions with nearly identical volume fraction of grains in both groups; (**b**) **Bimodal2**: two Gauss-type distributions with diverse volume fraction of grains in each group; (**c**) **Bimodal3**: microstructure consists of two lognormal distributions; (**d**) **Bimodal4**: grain assembly is composed of lognormal and Gauss-type distribution. The microstructural parameters are listed in Table 2. All microstructures are of the same scale.

Compared to the hypothetic bimodal distributions, real microstructures are more complex in the sense that each grain group reveals a certain size distribution. Figure 9 shows diverse synthetic microstructures, which are composed of larger and smaller grains; however, the type of grain size distribution is different in each case. The following bimodal distributions were studied: (i) Bimodal1: two Gauss-type distributions with nearly identical volume fractions of grains in both groups; (ii) Bimodal2: two Gauss distributions with diverse volume fractions of grains in each group; (iii) Bimodal3: two lognormal distributions; (iv) Bimodal4: lognormal–Gauss distribution. The characteristics of bimodal microstructures presented in Figure 9 are listed in Table 2 and Table 3. Although Bimodal1 is composed of two homogeneous grain groups, *H_v_*(large) = 0.996 and *H_v_*(small) = 0.965, the computed *H_v_*(Bimodal1) = 0.203 suggests that this microstructure is the most heterogeneous one compared to the counterparts presented in Figure 9b–d. Other computed indicators of homogeneity indicate a moderate degree of heterogeneity for the microstructure of Figure 9a (*H_d_*(Bimodal1) = 0.593, GI*(Bimodal1) = 0.493, HI*(Bimodal1) = 0.539); however, they seem to slightly overestimate the degree of homogeneity, as compared to the counterparts shown in Figure 9b–d. This is a consequence of the fact that the microstructure of Figure 9a discloses comparatively large values for both ratios *d_large_*/*d_small_* and *N_small_*/*N_large_* and as Figure 8 shows, *H_v_* tends to drop drastically when the volume fraction of large grains is low and the number of small grains is dominant. Further analysis of the quantitative indicators presented in Table 2 and Table 3 suggests that the quantitative measure of heterogeneity is also dependent on the type and combination of grain size distribution. Analyzing Table 3, it turns out that the correlation between the *H_v_* and *H_d_*, derived for the normal and lognormal distributions (see Figure 7b), does not apply to the bimodal microstructures. Similarly, the relationship developed for the GI* and HI* (Figure 7d) also does not follow the expected pattern due to the complex nature of bimodal grain assemblies.

## 4. Assessment of Experimentally Observed Microstructures and Texture Heterogeneity

**Figure 10:**
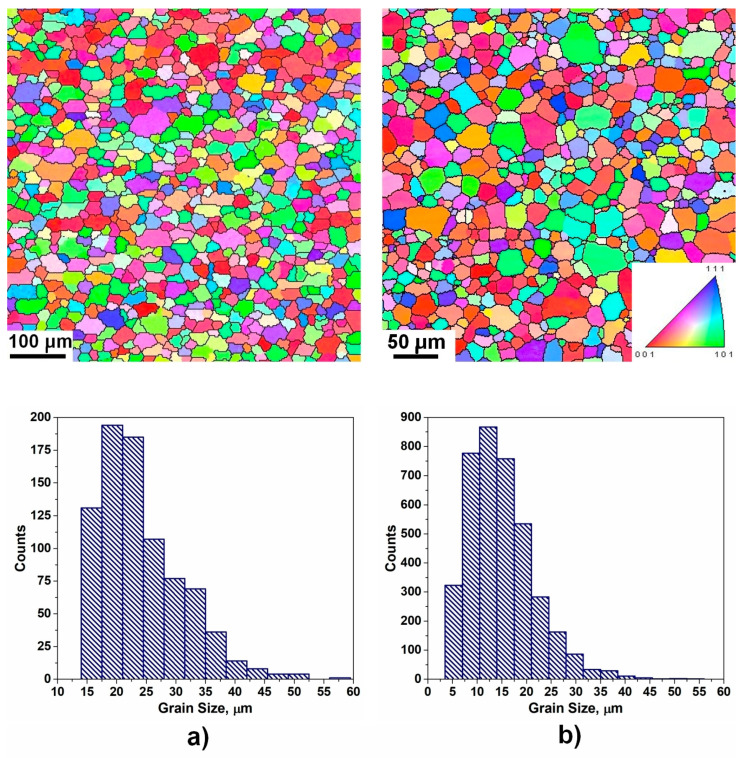
Microstructures measured by orientation image microscopy in aluminum alloys: (**a**) Al-Mg-Si alloy; (**b**) Al-Mg alloy (ND inverse pole figure maps are shown).

Figure 10a,b present microstructures measured by EBSD in two different Al alloys. Both polycrystalline aggregates show a lognormal size distribution. The computed quantitative indicators listed in Table 4 suggest that the microstructure of the investigated Al-Mg-Si alloy is more homogeneous compared to the Al-Mg counterpart. Both *H_v_* and GI* indicate that the microstructure of Figure 10a is of moderate heterogeneity, whereas the grain assembly shown in Figure 10b reveals microstructural characteristics of highly heterogeneous grain aggregate (see Table 3 and Table 4). By deconvoluting the grain size distributions of Figure 10 into two-grain groups (see Table 4), where the large grains in both cases reveal nearly identical values of *H_v_*, *H_d_*, GI*, and HI*, it becomes obvious that the heterogeneity is mainly caused by the broad spread of small grains. It can also be noticed that Figure 10b exhibits some distinctive features of bimodal distribution where the homogeneous group of large grains is mixed with the heterogeneous counterpart of a smaller grain size. Thus, it is shown here that the quantitative indicators employed for the assessment of microstructure homogeneity can be used to reveal the nature of complex microstructures.

The heterogeneity of the crystallographic texture in metallic materials might arise due to a number of reasons such as the following: (1) heterogeneity of the straining level and strain mode across the thickness caused by processing (rolling, extrusion, or friction stir welding [50]); (2) planar heterogeneity due to specific topological patterning of a particular texture component on the surface (roping phenomena [40,41]) or caused by TMP, where the texture in various volume elements differs [1]; and (3) local texture variations on the mesoscopic level due to localized strain or different recrystallization mechanisms [1,32].

The extent of texture severity can be expressed by the texture index *TI* and maximum value of ODF (*ODF_max_*), while the degree of deviation between macroscopic textures can be accessed by the normalized texture index difference expressed by equation 10. The texture gradient and angular deviation (see Equations (11) and (12)) allow the quantitative characterization of texture heterogeneity in a given direction. However, in many instances, these quantitative descriptions are not sufficient for the complete description of variations observed between two arbitrary textures.

In the case when TMP ensures homogeneous temperature, identical strain, and strain mode distribution in the whole volume of processed material (or one particular plane is analyzed), then qualitatively similar textures might evolve in the entire volume of the material (or investigated plane). For instance, the mid-thickness plane of rolled BCC metals (e.g., steel) is characterized by the {110}//RD and {111}//ND texture fibers, while {110}//ND and complex-shaped β-fiber [35] is observed in materials with an FCC crystal structure such as Al alloys [1,35,37]. In these cases, the degree of diversity for two textures *A* and *B* can be expressed by the normalized Pearson-type covariance (*R*) [10] of orientation densities along considered fibers:

(28) $$R=\frac{\sum_{i=1}^{n}\left({f\left(g\right)}_{i}^{A}-\bar{{f(g)}^{A}})({f\left(g\right)}_{i}^{B}-\bar{{f(g)}^{B}}\right)}{{\left(\sum_{i=1}^{n}{\left({f\left(g\right)}_{i}^{A}-\bar{{f(g)}^{A}}\right)}^{2}\sum_{i=1}^{n}{\left({f\left(g\right)}_{i}^{B}-\bar{{f(g)}^{B}}\right)}^{2}\right)}^{1/2}}$$

where the ${f\left(g\right)}_{i}^{X}$ values are the orientation densities along the considered fibers and $\bar{{f(g)}^{X}}$ is the average of the ${f\left(g\right)}_{i}^{X}$ dataset.

A high degree of homogeneity is reached when *R*^2^ approaches 1, implying that a strong correlation between the two counterparts is observed. In the case of weakly correlated datasets, the value of *R*^2^ is typically low. This type of analysis was carried out for the characterization of the crystallographic aspect of microstructure evolution during the equal channel angular process [51].

**Figure 11:**
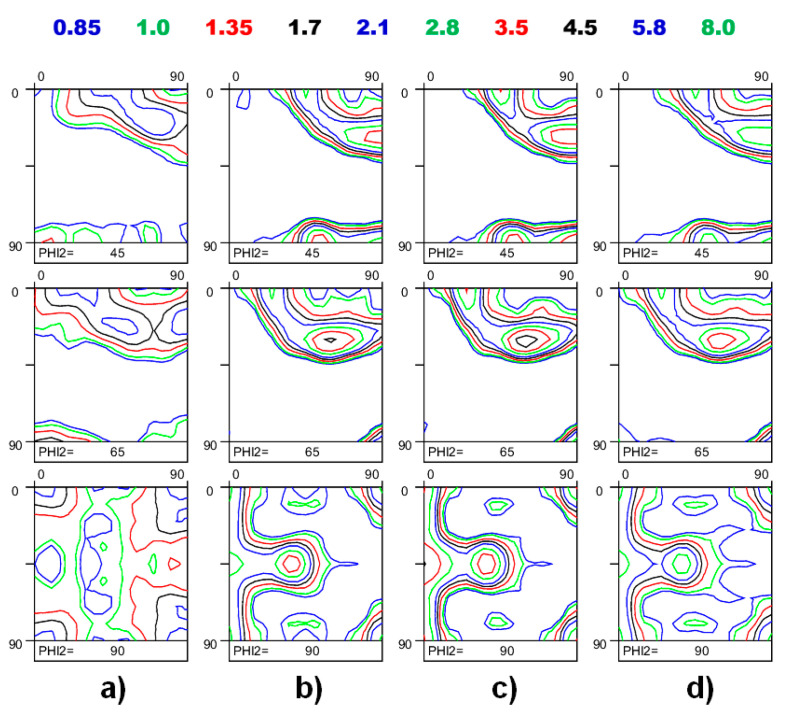
Evolution of deformation texture across the thickness of rolled Al-Mg-Si sheet: (**a**) surface texture; (**b**) subsurface texture; (**c**) mid-thickness texture; (**d**) overall texture.

Quite strong texture heterogeneity is typically observed across the thickness of industrially rolled metals due to inhomogeneous strain path evolution in each individual layer [1,52]. The unequal texture evolution across the thickness is induced by two competing shear components caused by the roll gap geometry and friction between the roll and the surface of the rolled sheet [52]. Figure 11 shows the ODFs of a rolled Al-Mg-Si sheet. The textures were measured on the surface, subsurface, and mid-thickness plane. The crystallographic textures of the examined layers show qualitative resemblance, whereas specific quantitative varieties can be observed between the individual counterparts. In analogy with Equation (10), the extent of texture heterogeneity (*TH*) between the individual layers *A* and *B* can be accessed as follows:

(29) $$TH=\frac{\int {\left({f(g)}^{A}-{f(g)}^{B}\right)}^{2}dg}{\int {\left({f(g)}^{O}\right)}^{2}dg}$$

where *f*(*g*)*^O^* refers to the overall texture (through-thickness texture).

It should be noted here that the analysis of texture heterogeneity by Equation (29) somewhat resembles the quantitative indicator expressed by Equation (11); however, instead of local texture variety observed between relatively tiny bands, Equation (29) characterizes changes between larger layers.

**Figure 12:**
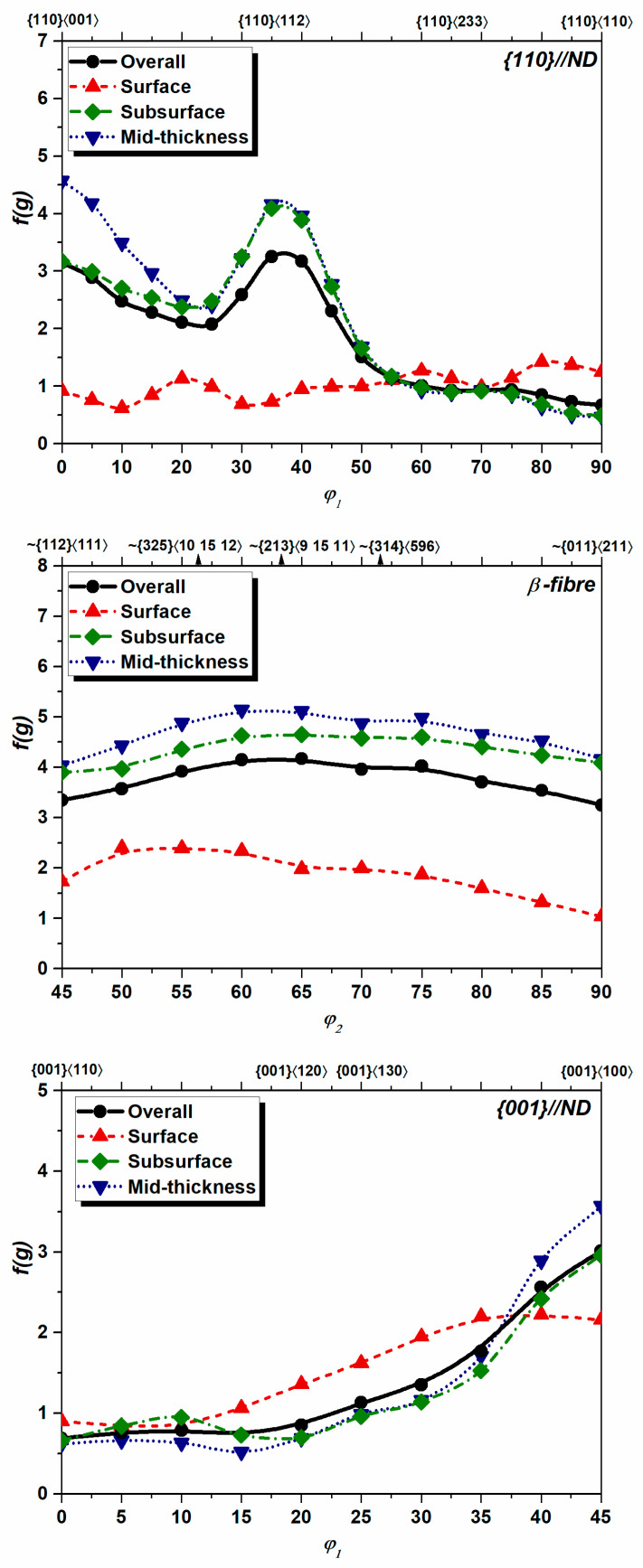
Distribution of texture density along the {110}//ND, {001}//ND, and β-fibers.

Analysis of the orientation densities along the {100}//ND, {110}//ND, and β-fibers (Figure 12) suggests that cold rolling has ensured evident texture diversity across the thickness. Table 5 shows the quantitative indicators of the texture assessment, which reveal that the degree of heterogeneity tends to drop from the surface towards mid-thickness. Analyzing both the *TH* and *ODF_max_* values listed in Table 5, the substantial differences in the surface and mid-thickness layers are not readily apparent due to the relatively low intensity of the overall texture (*ODF_max_*(overall) = 4.17). The low rate of *R*^2^ for the surface and mid-thickness layers (*R*^2^(surface–middle) = 0.114) reveals a weak correlation between the two textures evolved, whereas the neighboring layers show a higher degree of homogeneity (*R*^2^(surface–subsurface) = 0.741 and *R*^2^(subsurface–middle) = 0.968). This simple analysis exposes the nature of rolling and suggests that the subsurface and middle layers of rolled materials are subjected to more homogeneous straining as compared to the surface layers.

The number of grains in a representative volume element (RVE) is decisive for generating proper representative distributions. It is claimed that ~2700 grains seem to be sufficient for most microstructural parameters and correlations of measured grain distributions, each containing hundreds of grains per bin [44]. However, for most aspects of precise microstructure analysis, limitations tend to arise in examining correlations due to a relatively low grain count of coarse grains. Increasing the number of grains (e.g., doubling or tripling) in the RVE overcomes this constraint.

When it comes to the crystallographic aspects of a microstructure, a recent research endeavor [53] shows that the experimentally measured ODF of ~5400 grains is not always capable of reflecting all features of the texture evolved. In the study [53], the number of grains was correlated to the quality of crystal plasticity (CP) computations of the plastic strain ratio (Lankford value) and it was claimed that the accurate simulation requires information from ~20,000 experimentally measured grains [53]. Therefore, the large dataset containing information about the crystallographic orientation of grains in the arbitrary grain assembly is necessary to take into account the heterogeneity of both the microstructure and texture.

**Figure 13:**
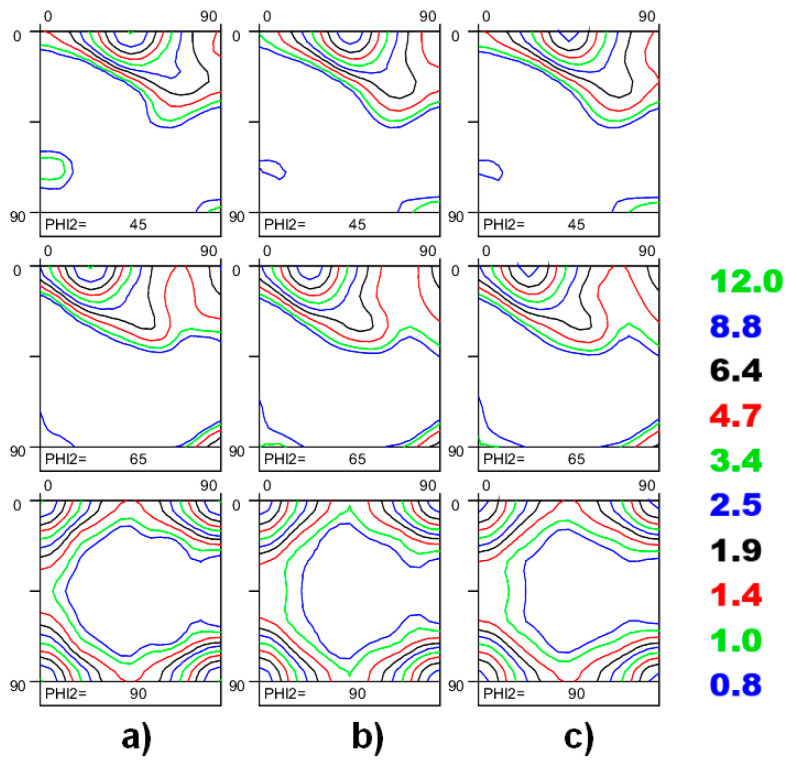
Texture diversity, caused by different grain statistics: (**a**) ODF composed of ~2000 grains; (**b**) ODF composed of ~8000 grains; (**c**) ODF composed of ~16,000 grains. The quantitative texture indicators are listed in Table 6.

**Figure 14:**
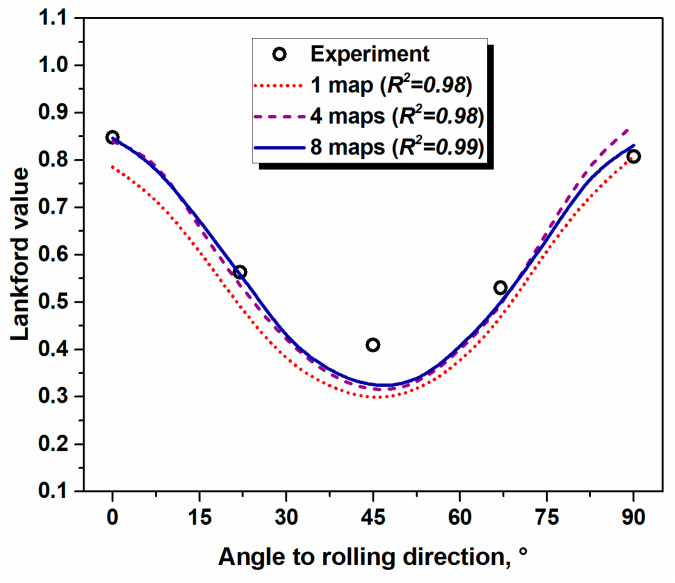
Experimentally measured and computed Lankford value profiles with various grain numbers (one OIM map contains ~2000 g) in 6xxx Al alloy.

Figure 13 shows the variety of textures evolved in Al-Mg-Si alloys. The samples were randomly selected from various locations of conventionally cold-rolled and heat-treated sheets of the following size ~300 mm × 300 mm with the aim of defining quantitative differences among the measured ODFs. Figure 13 shows the ODF of ~2000 grains (collected from a single EBSD scan, see Figure 13a), ~8000 grains (texture of four EBSD maps, see Figure 13b), and ~16,000 grains (texture of eight EBSD maps, see Figure 13c). By considering the texture of Figure 13c as a reference, the level of texture diversity can be estimated by Equation (10). The calculated *TID_N_* numbers, *TI*s, and *ODF_max_* values are listed in Table 6. The quantitative texture indicators computed for textures of Figure 13a–c indicate that a small degree of heterogeneity is observed in the investigated material. These minor texture deviations seem to have an impact on the quality of texture-based CP simulation (see Figure 14). Figure 14 reveals the quality of the CP simulations of the Lankford value by using the textures from Figure 13 as an input. It is obvious that the RVE composed of ~2000 crystals is not capable of ensuring the most accurate simulation of the plastic strain ratio, while using the texture computed from ~16,000 grains tends to improve the quality of the prediction (see Figure 14). Thus, it was shown here that the degree of texture heterogeneity has a decisive impact on the accuracy of texture-based simulations as well as on the performance of severely textured materials.

The results of the present investigation show that the degree of microstructural heterogeneity can be assessed by various methods. The derived *H_d_* and *H_v_* quantitative indicators seem to be suitable for the evaluation of heterogeneity. Depending on the application, the polycrystalline aggregate with a high degree of heterogeneity will have microvolumes that are different in terms of mechanical response (harder/softer, more/less ductile, etc.). The local microstructural varieties might affect the formability characteristics, wear resistance, or machinability. Therefore, it is important to control and evaluate the level of homogeneity in materials during thermomechanical processing routes. Furthermore, controlling the degree of microstructural heterogeneity can decrease the level of anisotropy, caused by the non-uniform micromechanical characteristics in the grain assembly. The results of the crystal plasticity simulations (Figure 14) reveal that the anisotropy of the plastic strain ratio, caused by the development of a crystallographic texture, can be reduced by strict texture control. Hence, the assessment of microstructural and crystallographic texture heterogeneity described in the frame of the present study is of crucial importance.

Modern materials characterization methods such as orientation imaging microscopy allow for analyzing microstructures in great detail. Furthermore, the experimentally measured data can be either exported to various postprocessing software [22,23] or, alternatively, synthetic microstructures with the features of experimental counterparts can be generated [43,44,45,46]. Analysis of virtual microstructures provides controlled environments for studying the mechanical/physical properties or performance of materials in various applications. Since the microstructural characteristics are digitalized, it is possible to compute the quantitative measures of heterogeneity, described in the frame of this work. Moreover, apart from the metallic microstructures, the polycrystalline aggregates of ceramics or polymers might be analyzed in the same manner.

## 5. Conclusions

1. The degree of microstructural homogeneity can be assessed by (i) relating the average grain size to the volume fraction weighted counterpart (*H_d_*), and (ii) relating the average grain volume to the volume fraction weighted equivalent (*H_v_*).
2. Both quantitative indicators of homogeneity (*H_d_* and *H_v_*) vary between 0 and 1. The highest degree of microstructural homogeneity is reached when *H_d_* ≈ 1 and *H_v_* ≈ 1, while in the case of highly heterogeneous grain assembly, both *H_d_* and *H_v_* tend to approach zero.
3. The derived quantitative measures of heterogeneity (*H_d_* and *H_v_*) correlate qualitatively with the measures of equality employed in economic statistics (Gini and Hoover indexes).
4. The results of the calculations suggest that in the case of bimodal distribution, *H_d_* and *H_v_* are dependent on the following parameters: (i) the grain size ratio of large to small grains; (ii) the grain number in either group; (iii) the volume fraction of grains in each group; (iv) and the type of grain size distribution of each grain group. Both quantitative indicators tend to drop drastically with an increase in the grain size ratio of large to small grains.
5. Even if the crystallographic textures show similar qualitative and quantitative features, the minor deviations caused by texture heterogeneity have a decisive impact on the texture-based simulation and texture-related properties in polycrystalline aggregates. Therefore, proper sampling and choice of the appropriate representative volume element is of crucial importance.

## Figures and Tables

**Table 1:** Quantitative indicators of various polycrystalline systems.

*d_a_*, μm	*d_r_*, μm	*std*	*H_d_*	*H_v_*	GI*	HI*	Figure
33.08	33.14	0.79	0.998	0.995	0.960	0.972	Figure 1a
41.41	112.63	24.79	0.368	0.066	0.225	0.394	Figure 1b
33.71	37.75	6.51	0.893	0.707	0.682	0.773	Figure 5a
36.41	57.42	14.52	0.634	0.257	0.412	0.562	Figure 5b
37.55	65.04	17.52	0.577	0.232	0.349	0.504	Figure 5c
149.02	153.71	15.28	0.969	0.912	0.829	0.878	Figure 5d
150.80	171.06	30.27	0.882	0.656	0.674	0.766	Figure 5e
154.51	184.76	37.57	0.836	0.572	0.608	0.716	Figure 5f
159.13	238.60	56.45	0.667	0.269	0.455	0.600	Figure 5g
163.89	250.35	63.83	0.655	0.298	0.422	0.569	Figure 5h
12.20	12.61	1.29	0.968	0.907	0.823	0.874	Figure 5i
13.57	22.03	5.44	0.616	0.232	0.402	0.554	Figure 5j

**Table 2:** Quantitative characteristics of bimodal microstructures.

	*d_a_*, μm	*d_r_*, μm	*std*	*d_large_*/*d_small_*	*N_small_*/*N_large_*	*w*	Figure
**Bimodal1**	18.36	30.93	6.38	2.59	16.34	-	Figure 9a
large grains	43.83	43.89	0.92		-	0.52	
small grains	16.80	16.97	0.97		-	0.48	
**Bimodal2**	21.96	38.20	10.30	2.48	4.76	-	Figure 9b
large grains	44.16	44.20	0.80		-	0.77	
small grains	17.30	17.80	1.70		-	0.23	
**Bimodal3**	22.80	40.61	11.04	2.39	4.32	-	Figure 9c
large grains	44.21	46.80	6.13		-	0.77	
small grains	17.84	19.60	3.22		-	0.23	
**Bimodal4**	22.57	38.46	10.81	2.51	4.25	-	Figure 9d
large grains	44.02	44.10	0.98		-	0.77	
small grains	17.56	19.32	3.18		-	0.23	

**Table 3:** Quantitative indicators of homogeneity computed for bimodal microstructures.

	*H_d_*	*H_v_*	GI*	HI*	Figure
**Bimodal1**	0.593	0.203	0.493	0.539	Figure 9a
**Bimodal2**	0.575	0.284	0.366	0.401	Figure 9b
**Bimodal3**	0.561	0.260	0.328	0.416	Figure 9c
**Bimodal4**	0.587	0.309	0.358	0.417	Figure 9d

**Table 4:** Microstructural characteristics of investigated Al alloys.

*d_a_*, μm	*d_r_*, μm	*std*	*H_d_*	*H_v_*	GI*	HI*	Comment	Figure
24.15	31.03	7.05	0.778	0.478	0.535	0.652	all g	Figure 10a
22.77	26.42	5.31	0.862	0.669	0.624	0.719	small g	
39.95	41.90	4.77	0.953	0.851	0.804	0.901	large g	
14.90	24.28	6.62	0.614	0.268	0.381	0.538	all g	Figure 10b
14.46	20.93	5.85	0.691	0.411	0.434	0.576	small g	
37.03	38.87	4.41	0.953	0.848	0.805	0.857	large g	

**Table 5:** Texture indicators of rolled Al-Mg-Si alloy.

Layer	*ODF_max_*	*TI*
**Surf**	2.77	1.285
**Subs**	4.66	1.844
**Mid**	5.14	2.031
**Overall**	4.17	1.654
**Layers**	***R* ^2^**	***TH***
**Surf**–**subs**	0.741	0.336
**Subs**–**middle**	0.968	0.185
**Surf**–**middle**	0.114	0.439

**Table 6:** Quantitative texture characteristics of OIM maps, consisting of various grain numbers.

Texture	*ODF_max_*	*TI*	*TID_n_*
Figure 13a (~2000 grains)	12.34	2.301	2.0 × 10^−2^
Figure 13b (~8000 grains)	10.79	2.101	3.2 × 10^−3^
Figure 13c (~16,000 grains)	9.96	1.982	reference

## Data Availability

Data are currently used in various postprocessing procedures and can be shared upon request on collaborative basis.
